# Supplementary material for: Effect of remdesivir post hospitalization for COVID-19 infection from the randomized SOLIDARITY Finland trial
Source: Nat Commun. 2022 Oct 18;13:6152. doi: 10.1038/s41467-022-33825-5 (PMC9579198; doi:10.1038/s41467-022-33825-5)
Supplement: Supplementary file 1 — Supplementary Information [file 41467_2022_33825_MOESM1_ESM.pdf]

# Supplement

Effect of remdesivir post hospitalization for COVID-19 infection from the randomized SOLIDARITY Finland trial

**Supplementary Table 1**. EQ-5D-5L responses in long-term follow-up of patients who received remdesivir in the acute phase of COVID-19 infection (pages 2-3)

**Supplementary Table 2**. Dichotomized EQ-5D-5L responses as analysed (page 4)

**Supplementary Table 3.** Symptoms after one year of hospitalization between treatment arms (pages 5-7)

**Supplementary Table 4**. The effect of remdesivir plus standard of care compared to standard of care only on self-reported symptoms after one year from hospitalization due to COVID-19 infection (page 8)

**Supplementary Table 5**. The effect of remdesivir plus standard of care compared to standard of care only on recovery, stratified by the need of oxygen therapy during hospital admission (page 9)

**Code** (page 9)

| **Supplementary Table 1**. EQ-5D-5L responses in long-term follow-up of patients who received remdesivir in the acute phase of COVID-19 infection | | | |
|---|---|---|---|
| | Remdesivir, n=98 (%) | SoC, n=83 (%) | Both, n=181 (%) |
| **Mobility** | | | |
| 1) No problems | 58 (59.2) | 56 (67.5) | 114 (63) |
| 2) Slight problems | 23 (23.5) | 13 (15.7) | 36 (19.9) |
| 3) Moderate problems | 10 (10.2) | 8 (9.6) | 18 (9.9) |
| 4) Severe problems | 5 (5.1) | 6 (7.2) | 11 (6.1) |
| 5) Unable to walk | 2 (2.0) | 0 | 2 (1.1) |
| **Self-care** | | | |
| 1) No problems | 89 (90.8) | 73 (88.0) | 162 (89.5) |
| 2) Slight problems | 6 (6.1) | 5 (6.0) | 11 (6.1) |
| 3) Moderate problems | 0 | 4 (4.8) | 4 (2.2) |
| 4) Severe problems | 2 (2.0) | 1 (1.2) | 3 (1.7) |
| 5) Unable to wash or dress | 1 (1.0) | 0 | 1 (0.6) |
| **Usual activities** | | | |
| 1) No problems | 66 (67.3) | 57 (68.7 | 123 (68.0) |
| 2) Slight problems | 22 (22.4) | 14 (16.9) | 36 (19.9) |
| 3) Moderate problems | 6 (6.1) | 6 (7.2) | 12 (6.6) |
| 4) Severe problems | 4 (4.1) | 6 (7.2) | 10 (5.5) |
| 5) Unable to do usual activities | 0 | 0 | 0 |
| **Pain / discomfort** | | | |
| 1) No pain | 48 (49) | 35 (42.2) | 83 (45.9) |
| 2) Slight pain | 35 (35.7) | 33 (39.8) | 68 (37.6) |
| 3) Moderate pain | 11 (11.2) | 11 (13.3) | 22 (12.2) |
| 4) Severe pain | 2 (2.0) | 3 (3.6) | 5 (2.8) |
| 5) Extreme pain | 2 (2.0) | 1 (1.2) | 3 (1.7) |
| **Anxiety / depression** | | | |
| 1) No | 65 (66.3) | 57 (68.7) | 122 (67.4) |
| 2) Slight | 24 (24.5) | 20 (24.1) | 44 (24.3) |
| 3) Moderate | 7 (7.1) | 3 (3.6) | 10 (5.5) |

| | | | |
|---|---|---|---|
| 4) Severe | 1 (1.0) | 3 (3.6) | 4 (2.2) |
| 5) Extreme | 1 (1.0) | 0 | 1 (0.6) |

- EQ-5D, mobility: One patient reported both 3-4 and was classified as 4. One patient reported both 1-2 and was classified as 2.
- EQ-5D, self-care: One patient reported both 3-4 and was treated as 4.
- EQ-5D, pain: One patient reported both 1-2 and was classified as 2.

| **Supplementary Table 2**. Dichotomized EQ-5D-5L responses as analysed | | | |
|---|---|---|---|
| | Remdesivir, n=98 (%) | SoC, n=83 (%) | Both, n=181 (%) |
| **Mobility** | | | |
| Outcomes 1-2 | 81 (82.7) | 69 (83.1) | 150 (82.9) |
| Outcomes 3-5 | 17 (17.3) | 14 (16.9) | 31 (17.1) |
| **Self-care** | | | |
| Outcomes 1-2 | 95 (96.9) | 78 (94) | 173 (95.6) |
| Outcomes 3-5 | 3 (3.1) | 5 (6.0) | 8 (4.4) |
| **Usual activities** | | | |
| Outcomes 1-2 | 88 (89.7) | 71 (85.5) | 159 (87.8) |
| Outcomes 3-5 | 10 (10.2) | 12 (14.5) | 22 (12.2) |
| **Pain /discomfort** | | | |
| Outcomes 1-2 | 83 (84.7) | 68 (81.9) | 151 (83.4) |
| Outcomes 3-5 | 15 (15.3) | 15 (18.1) | 30 (16.6) |
| **Anxiety /depression** | | | |
| Outcomes 1-2 | 89 (90.8) | 77 (92.8) | 166 (91.7) |
| Outcomes 3-5 | 9 (9.1) | 6 (7.2) | 15 (8.3) |

| **Supplementary Table 3.** Symptoms after one year of hospitalization between treatment arms | | | | |
|---|---|---|---|---|
| | No symptom (0) | Mild symptom (1) | Moderate symptom (2) | Severe symptom (3) |
| **Temperature** | | | | |
| - Remdesivir, n (%) | 91 (92.9) | 7 (7.1) | 0 | 0 |
| - Standard of care, n (%) | 77 (92.8) | 6 (7.2) | 0 | 0 |
| - Total, n (%) | 168 (92.8) | 13 (7.2) | 0 | 0 |
| **Fatigue** | | | | |
| - Remdesivir, n (%) | 42 (42.9) | 32 (32.7) | 17 (17.3) | 7 (7.1) |
| - Standard of care, n (%) | 37 (44.6) | 23 (27.7) | 13 (15.7) | 10 (12.0) |
| - Total, n (%) | 79 (43.6) | 55 (30.4) | 30 (16.6) | 17 (9.4) |
| **Problems with attention and concentration** | | | | |
| - Remdesivir, n (%) | 50 (51) | 30 (30.6) | 8 (8.2) | 10 (10.2) |
| - Standard of care, n (%) | 47 (56.6) | 21 (25.3) | 9 (10.8) | 6 (7.2) |
| - Total, n (%) | 97 (53.6) | 51 (28.2) | 17 (9.4) | 16 (8.8) |
| **Memory difficulties and forgetting things** | | | | |
| - Remdesivir, n (%) | 45 (45.9) | 32 (32.7) | 13 (13.3) | 8 (8.2) |
| - Standard of care, n (%) | 41 (49.4) | 28 (33.7) | 11 (13.3) | 3 (3.6) |
| - Total, n (%) | 86 (47.5) | 60 (33.1) | 24 (13.3) | 11 (6.1) |
| **Difficulty sleeping or falling asleep** | | | | |
| - Remdesivir, n (%) | 52 (53.1) | 32 (32.7) | 6 (6.1) | 8 (8.2) |
| - Standard of care, n (%) | 53 (63.9) | 14 (16.9) | 11 (13.3) | 5 (6.0) |
| - Total, n (%) | 105 (58.0) | 46 (25.4) | 17 (9.4) | 13 (7.2) |
| **Depression, low spirits** | | | | |
| - Remdesivir, n (%) | 66 (67.3) | 20 (20.4) | 9 (9.2) | 3 (3.1) |
| - Standard of care, n (%) | 50 (60.2) | 22 (26.5) | 6 (7.2) | 5 (6.0) |
| - Total, n (%) | 116 (64.1) | 42 (23.2) | 15 (8.3) | 8 (4.4) |
| **Mental anxiety, nervousness** | | | | |
| - Remdesivir, n (%) | 65 (66.3) | 21 (21.4) | 9 (9.2) | 3 (3.1) |
| - Standard of care, n (%) | 59 (71.1) | 13 (15.7) | 7 (8.4) | 4 (4.8) |
| - Total, n (%) | 124 (68.5) | 34 (18.8) | 16 (8.8) | 7 (3.9) |

| | | | | |
|---|---|---|---|---|
| **Dizziness, balance problems** | | | | |
| - Remdesivir, n (%) | 70 (71.4) | 21 (21.4) | 5 (5.1) | 2 (2.0) |
| - Standard of care, n (%) | 57 (68.7) | 20 (24.1) | 0 | 6 (7.2) |
| - Total, n (%) | 127 (70.2) | 41 (22.7) | 5 (2.8) | 8 (4.4) |
| **Headache** | | | | |
| - Remdesivir, n (%) | 70 (71.4) | 20 (20.4) | 5 (5.1) | 3 (3.1) |
| - Standard of care, n (%) | 60 (72.3) | 14 (16.9) | 9 (10.8) | 0 |
| - Total, n (%) | 130 (71.8) | 34 (18.8) | 14 (7.7) | 3 (1.7) |
| **Tinnitus** | | | | |
| - Remdesivir, n (%) | 72 (73.5) | 15 (15.3) | 7 (7.1) | 3 (3.1) |
| - Standard of care, n (%) | 59 (71.1) | 19 (22.9) | 4 (4.8) | 1 (1.2) |
| - Total, n (%) | 131 (72.4) | 34 (18.8) | 11 (6.1) | 4 (2.2) |
| **[a]Skin sensation disorders and tingling** | | | | |
| - Remdesivir, n (%) | 70 (71.4) | 18 (18.4) | 5 (5.1) | 5 (5.1) |
| - Standard of care, n (%) | 61 (73.5) | 17 (20.5) | 5 (6.0) | 0 |
| - Total, n (%) | 131 (72.4) | 35 (19.3) | 10 (5.5) | 5 (2.8) |
| **Change in the sense of smell and/or taste** | | | | |
| - Remdesivir, n (%) | 76 (77.6) | 13 (13.3) | 6 (6.1) | 3 (3.1) |
| - Standard of care, n (%) | 63 (75.9) | 16 (19.3) | 3 (3.6) | 1 (1.2) |
| - Total, n (%) | 139 (76.8) | 29 (16.0) | 9 (5.0) | 4 (2.2) |
| **Prolonged general malaise following even light exertion** | | | | |
| - Remdesivir, n (%) | 79 (80.6) | 14 (14.3) | 3 (3.1) | 2 (2.0) |
| - Standard of care, n (%) | 63 (75.9) | 9 (10.8) | 8 (9.6) | 3 (3.6) |
| - Total | 142 (78.5) | 23 (12.7) | 11 (6.1) | 5 (2.8) |
| **Cardiac arrhythmias or increased resting heart rate** | | | | |
| - Remdesivir, n (%) | 66 (67.3) | 24 (24.5) | 6 (6.1) | 2 (2.0) |
| - Standard of care, n (%) | 57 (68.7) | 16 (19.3) | 7 (8.4) | 3 (3.6) |
| - Total, n (%) | 123 (68.0) | 40 (22.1) | 13 (7.2) | 5 (2.8) |
| **A feeling of pressure or discomfort in the chest** | | | | |
| - Remdesivir, n (%) | 69 (70.4) | 23 (23.5) | 4 (4.1) | 2 (2.0) |

| | | | | |
|---|---|---|---|---|
| - Standard of care, n (%) | 66 (79.5) | 10 (12.0) | 5 (6.0) | 2 (2.4) |
| - Total | 135 (74.6) | 33 (18.2) | 9 (5.0) | 4 (2.2) |
| **Nausea, queasiness** | | | | |
| - Remdesivir, n (%) | 85 (86.7) | 11 (11.2) | 2 (2.0) | 0 |
| - Standard of care, n (%) | 72 (86.7) | 10 (12.0) | 1 (1.2) | 0 |
| - Total, n (%) | 157 (86.7) | 21 (11.6) | 3 (1.7) | 0 |
| **Rash** | | | | |
| - Remdesivir, n (%) | 82 (83.7) | 13 (13.3) | 2 (2.0) | 1 (1.) |
| - Standard of care, n (%) | 57 (68.7) | 21 (25.3) | 4 (4.8) | 1 (1.2) |
| - Total, n (%) | 139 (76.8) | 34 (18.8) | 6 (3.3) | 2 (1.1) |
| **Joint pain** | | | | |
| - Remdesivir, n (%) | 55 (56.1) | 20 (20.4) | 18 (18.4) | 5 (5.1) |
| - Standard of care, n (%) | 45 (54.2) | 21 (25.3) | 14 (16.9) | 3 (3.6) |
| - Total | 100 (55.2) | 41 (22.7) | 32 (17.7) | 8 (4.4) |
| **Muscle pain** | | | | |
| - Remdesivir, n (%) | 60 (61.2) | 27 (27.6) | 8 (8.2) | 3 (3.1) |
| - Standard of care, n (%) | 48 (57.8) | 22 (26.5) | 12 (14.5) | 1 (1.2) |
| - Total, n (%) | 181 (59.7) | 49 (27.1) | 20 (11.0) | 4 (2.2) |
| **Persistent weekly cough** | | | | |
| - Remdesivir, n (%) | 72 (73.5) | 13 (13.3) | 12 (12.2) | 1 (1.0) |
| - Standard of care, n (%) | 62 (74.7) | 9 (10.8) | 9 (10.8) | 3 (3.6) |
| - Total, n (%) | 134 (74.0) | 22 (12.2) | 21 (11.6) | 4 (2.2) |
| **Persistent weekly respiratory mucus** | | | | |
| - Remdesivir, n (%) | 68 (69.4) | 8 (8.2) | 20 (20.4) | 2 (2.0) |
| - Standard of care, n (%) | 51 (61.4) | 16 (19.3) | 13 (15.7) | 3 (3.6) |
| - Total, n (%) | 119 (65.7) | 24 (13.3) | 33 (18.2) | 5 (2.8) |
| [a]With skin rash, one patient, selected both 0 and 1, and is coded as 1. | | | | |

| **Supplementary Table 4**. The effect of remdesivir plus standard of care compared to standard of care only on self-reported symptoms after one year from hospitalization due to COVID-19 infection | | |
|---|---|---|
| | Unadjusted odds ratio | 95% confidence interval |
| **Temperature** | NA | NA |
| **Problems with attention and concentration** | 1.02 | 0.55-1.89 |
| **Memory difficulties and forgetting things** | 1.27 | 0.69-2.34 |
| **Difficulty sleeping or falling asleep** | 0.74 | 0.39-1.43 |
| **Depression, low spirits** | 0.92 | 0.43-1.98 |
| **Mental anxiety, nervousness** | 0.92 | 0.43-1.98 |
| **Dizziness, balance problems** | 0.99 | 0.35-2.83 |
| **Headache** | 0.75 | 0.30-1.86 |
| **Tinnitus, i.e., ringing in the ear** | 1.71 | 0.61-4.81 |
| **Skin sensation disorders and tingling** | 1.69 | 0.61-4.76 |
| **Change in the sense of smell and/or taste** | 1.91 | 0.61-5.96 |
| **Prolonged general malaise following even light exertion** | 0.39 | 0.14-1.06 |
| **Cardiac arrhythmias or increased resting heart rate** | 0.68 | 0.28-1.64 |
| **A feeling of pressure or discomfort in the chest** | 0.73 | 0.25-2.08 |
| **Nausea, queasiness** | 1.69 | 0.16-19.18 |
| **Rash** | 0.51 | 0.13-2.06 |
| **Joint pain** | 1.15 | 0.66-2.00 |
| **Muscle pain** | 0.72 | 0.34-1.51 |
| **Persistent weekly cough** | 0.92 | 0.44-1.90 |
| **Persistent weekly respiratory mucus** | 1.16 | 0.66-2.07 |

| **Supplementary Table 5**. The effect of remdesivir plus standard of care compared to standard of care only on recovery, stratified by the need of oxygen therapy during hospital admission | | |
|---|---|---|
| | Relative risk | 95% confidence interval |
| **Recovery in patients without oxygen during admission** | 0.88 | 0.65-1.18 |
| **Recovery in patients with oxygen during admission** | 1.03 | 0.90-1.17 |

## Code

Statistical analysis was performed using RStudio. Binary outcomes were treated in a similar fashion as mortality at one year as shown below. The groups were standard of care (0) and standard of care plus remdesivir (1). The Epi-package was used.

library(Epi)

tmp.tab<-with(Mortality1y,t(table(Death=factor(Death1v,levels = 1:0,labels = c("dead","alive")), Group=factor(Group,levels = 1:0) )))

twoby2(exposure=(tmp.tab))

```
## 2 by 2 table analysis:
## ------------------------------------------------------
## Outcome   : dead
## Comparing : 1 vs. 0
##
##   dead alive    P(dead) 95% conf. interval
## 1    5   109     0.0439    0.0184   0.1011
## 0    5    89     0.0532    0.0223   0.1215
##
##                                   95% conf. interval
##              Relative Risk:  0.8246    0.2461   2.7628
##          Sample Odds Ratio:  0.8165    0.2291   2.9099
## Conditional MLE Odds Ratio:  0.8173    0.1819   3.6725
##     Probability difference: -0.0093   -0.0793   0.0532
##
##              Exact P-value: 0.7575
##         Asymptotic P-value: 0.7546
## ------------------------------------------------------
```
