# Supplementary material for: Effect of remdesivir post hospitalization for COVID-19 infection from the randomized SOLIDARITY Finland trial
Source: Nat Commun. 2022 Oct 18;13:6152. doi: 10.1038/s41467-022-33825-5 (PMC9579198; doi:10.1038/s41467-022-33825-5)
Supplement: Supplementary file 3 — Reporting Summary [file 41467_2022_33825_MOESM3_ESM.pdf]

nature portfolio

Corresponding author(s): Kari Tikkinen
Last updated by author(s): Sep 19, 2022

Reporting Summary

Nature Portfolio wishes to improve the reproducibility of the work that we publish. This form provides structure for consistency and transparency in reporting. For further information on Nature Portfolio policies, see our Editorial Policies and the Editorial Policy Checklist.

Statistics

For all statistical analyses, confirm that the following items are present in the figure legend, table legend, main text, or Methods section.

n/a	Confirmed	
☐	☒	The exact sample size (*n*) for each experimental group/condition, given as a discrete number and unit of measurement
☐	☒	A statement on whether measurements were taken from distinct samples or whether the same sample was measured repeatedly
☐	☒	The statistical test(s) used AND whether they are one- or two-sided<br>*Only common tests should be described solely by name; describe more complex techniques in the Methods section.*
☐	☒	A description of all covariates tested
☐	☒	A description of any assumptions or corrections, such as tests of normality and adjustment for multiple comparisons
☐	☒	A full description of the statistical parameters including central tendency (e.g. means) or other basic estimates (e.g. regression coefficient) AND variation (e.g. standard deviation) or associated estimates of uncertainty (e.g. confidence intervals)
☐	☒	For null hypothesis testing, the test statistic (e.g. *F*, *t*, *r*) with confidence intervals, effect sizes, degrees of freedom and *P* value noted<br>*Give P values as exact values whenever suitable.*
☒	☐	For Bayesian analysis, information on the choice of priors and Markov chain Monte Carlo settings
☒	☐	For hierarchical and complex designs, identification of the appropriate level for tests and full reporting of outcomes
☒	☐	Estimates of effect sizes (e.g. Cohen's *d*, Pearson's *r*), indicating how they were calculated

Our web collection on statistics for biologists contains articles on many of the points above.

Software and code

Policy information about availability of computer code

Data collection	Castor EDC software
Data analysis	RStudio, version 1.4.1106

For manuscripts utilizing custom algorithms or software that are central to the research but not yet described in published literature, software must be made available to editors and reviewers. We strongly encourage code deposition in a community repository (e.g. GitHub). See the Nature Portfolio guidelines for submitting code & software for further information.

Data

Policy information about availability of data

All manuscripts must include a data availability statement. This statement should provide the following information, where applicable:

- Accession codes, unique identifiers, or web links for publicly available datasets
- A description of any restrictions on data availability
- For clinical datasets or third party data, please ensure that the statement adheres to our policy

Detailed aggregate level data is available in the online Supplement. The dataset generated during and analyzed during the current study are not publicly available. The corresponding author (K.A.O.T.) is the custodian of the data and will provide access to de-identified and processed participant data for academic purposes on request (kari.tikkinen@helsinki.fi), with the completion of a data access agreement. The reason for controlled access is that we are yet in the process of creating another article partly based on this same data.

Human research participants

Policy information about studies involving human research participants and Sex and Gender in Research.

Reporting on sex and gender	Both men and women were recruited for the trial (and the follow-up). As typical in COVID, there were more men than women in hospitalized patients (64% of the recruited were men).
Population characteristics	Adult patient (≥18 years) hospitalized due to COVID. Mean age was 58.3 years (SD 13.4; range 25-88 years).
Recruitment	Patients with PCR-confirmed COVID-19 requiring hospitalization were recruited from 11 Finnish hospital. During the study period, of those patients who were treated for COVID more than 24 hours in these hospitals, more than 23% were recruited to our trial. This suggests that recruitment process worked very well. We used randomization to prevent imbalance of prognostic factors (for comparisons of treatment effect).
Ethics oversight	Helsinki University Hospital Ethics board approved the study (HUS/1866/2021)

Note that full information on the approval of the study protocol must also be provided in the manuscript.

Field-specific reporting

Please select the one below that is the best fit for your research. If you are not sure, read the appropriate sections before making your selection.

☒ Life sciences ☐ Behavioural & social sciences ☐ Ecological, evolutionary & environmental sciences

For a reference copy of the document with all sections, see nature.com/documents/nr-reporting-summary-flat.pdf

Life sciences study design

All studies must disclose on these points even when the disclosure is negative.

Sample size	The SOLIDARITY Finland trial (in which patients were originally recruited) is part of the global WHO-led study; no sample size calculations for individual countries. Patient recruitment was halted according to the WHO instructions, when the global SOLIDARITY had gathered enough patients. SOLIDARITY Finland is therefore underpowered to detect small effects. However, our trial is an important starting point for the evidence-base of long-term follow-ups of COVID-19 drug treatments/trials.
Data exclusions	At recruiment, we excluded patients who had an estimated life expectancy of <3 months, another acute severe condition during the past week, liver enzyme levels more than five-fold over the upper reference limit, severe kidney failure, or who were pregnant or breastfeeding, or participated in another trial. All patients were invited to participate in the long-term follow-up.
Replication	Main analyses were replicated by two separate people (both authors of the article) and critically reviewed by the team.
Randomization	We used web-based program CastorEDC to randomize participants (1:1)
Blinding	Open-label study; large, simple trial in hospitals stretched due to pandemic.

Reporting for specific materials, systems and methods

We require information from authors about some types of materials, experimental systems and methods used in many studies. Here, indicate whether each material, system or method listed is relevant to your study. If you are not sure if a list item applies to your research, read the appropriate section before selecting a response.

Materials & experimental systems

n/a	Involved in the study	
☒	☐	Antibodies
☒	☐	Eukaryotic cell lines
☒	☐	Palaeontology and archaeology
☒	☐	Animals and other organisms
☐	☒	Clinical data
☒	☐	Dual use research of concern

Methods

n/a	Involved in the study	
☒	☐	ChIP-seq
☒	☐	Flow cytometry
☒	☐	MRI-based neuroimaging

Clinical data

Policy information about clinical studies

All manuscripts should comply with the ICMJE guidelines for publication of clinical research and a completed CONSORT checklist must be included with all submissions.

Clinical trial registration	ClinicalTrials.gov NCT04978259
Study protocol	Study protocol is as a supplement of this submission.
Data collection	We sent questionnaires via post mail to patients who participated the trial (from 11 Finnish hospitals) in its hospital phase one year earlier between July 2021 and January 2022. A remainder post was sent, and if necessary, the patient was also contacted via telephone.
Outcomes	Outcomes (as listed in ClinicalTrials) included recovery, quality of life, dyspnea, fatigue and other potential long-COVID symptoms that were obtained via (mailed) questionnaire. Mortality was obtained from national database (DVV).
